# Normalized difference vegetation index, temperature and age affect faecal thyroid hormone concentrations in free-ranging African elephants

**DOI:** 10.1093/conphys/coaa010

**Published:** 2020-04-04

**Authors:** Isabelle D Szott, Yolanda Pretorius, Andre Ganswindt, Nicola F Koyama

**Affiliations:** 1 School of Biological and Environmental Sciences, Liverpool John Moores University, Liverpool L3 3AF, UK; 2 Mammal Research Institute, University of Pretoria, Hatfield 0028, South Africa; 3 Southern African Wildlife College, Hoedspruit 1380, South Africa; 4 Endocrine Research Laboratory, Department of Anatomy and Physiology, University of Pretoria, Onderstepoort 0110, South Africa

**Keywords:** Animal welfare, conservation, endocrine marker, *Loxodonta africana*, non-invasive hormone monitoring

## Abstract

Conservation biologists can use hormone measurements to assess animals’ welfare, reproductive state, susceptibility to stressors, as well as energy expenditure. Quantifying hormone concentrations from faecal samples is particularly advantageous as samples can be collected without disturbing animals’ behaviour. In order for an endocrine marker to be useful for wildlife managers, we need to understand how extrinsic and intrinsic factors affect hormone concentrations in free-ranging animal populations. Thyroid hormones are linked to basal metabolic rate and energy expenditure. Previous research demonstrated that triiodothyronine (T3) can be measured successfully in faecal matter of African elephants, *Loxodonta africana*. However, to our knowledge, research into factors affecting changes in elephant T3 levels has only been carried out in captive elephants so far. Thus, we present the first study of faecal T3 metabolite (mT3) concentrations of a large population of free-ranging African elephants. Over 15 months, we collected faecal samples from identified (*n* = 43 samples) and unidentified (*n* = 145 samples) individuals in Madikwe Game Reserve, South Africa. We investigated whether vegetative productivity [normalized difference vegetation index (NDVI)] in interaction with mean monthly temperature, age and sex affected mT3 concentrations. We found a significant negative interaction effect of NDVI and temperature. Increasing NDVI was related to higher concentrations of mT3, but increasing temperature was related to a decrease in mT3 concentrations in individually identified and unidentified elephants. In unidentified individuals, juvenile elephants had significantly higher mT3 concentrations compared to adult elephants. Faecal T3 can successfully be quantified in samples from free-ranging elephant populations and thus provides insight into energy expenditure in large herbivores.

## Introduction

Monitoring of hormones of wild and captive animals has long been used by conservation biologists to investigate areas of interest, including animal health and welfare and how individuals cope with environmental challenges (e.g. Millspaugh and Washburn, 2004; Millspaugh *et al*., 2007; Teixeira *et al*., 2007; Ganswindt *et al*., 2010a, b; Palme, 2012; Scheun *et al*., 2015). According to the energy allocation model, energy can be allocated to reproduction, somatic growth and maintenance or storage (Perrin and Sibly, 1993; Roff and Fairbairn, 2007; French *et al*., 2009). Through the assessment of energetic condition conservation biologists can infer the effects of changing environmental conditions and anthropogenic disturbance on an animal’s energy budget, behaviour and life history (Ellenberg *et al*., 2007; Schaebs *et al*., 2016; Gesquiere *et al*., 2018). This is especially important in species with slow growth rates and reproduction (Schaebs *et al*., 2016).

Thyroid hormones are closely linked to energy balance (Eales, 1988; Behringer *et al*., 2018; Gesquiere *et al*., 2018) and can reflect resource limitation in populations of wild animals (Gobush *et al*., 2014), making them a useful endocrine marker for wildlife conservation and management. Thyroid hormones increase adenosine triphosphate production for metabolic processes such as activity, excess intake of calories, fever or changes in environmental temperature and therefore correlate with basal metabolic rate (BMR) in terms of lean body mass and energy expenditure (Bianco *et al*., 2005; Mullur *et al*., 2014). Reduced caloric intake is linked to a decrease in thyroid hormone concentrations and reduced BMR, while greater energy expenditure requires an increase in BMR, which is linked to increased thyroid hormone concentrations [e.g. howler monkeys, *Alouatta palliata* (Wasser *et al*., 2010; Dias *et al*., 2017), Barbary macaques, *Macaca sylvanus* (Cristóbal-Azkarate *et al*., 2016); yellow-breasted capuchins, *Sapajus xanthosternos* (Schaebs *et al*., 2016), baboons, *Papio* spp. (Gesquiere *et al*., 2018); Eales, 1988; Behringer *et al*., 2018]. Varying thyroid hormone concentrations are extremely important for free-living mammals because they allow adaptation of metabolic balance to variations in environmental conditions, nutrient requirements and availability, as well as homeoretic changes during different physiological stages (Todini *et al*., 2007; Behringer *et al*., 2018). Recently, Behringer *et al*. (2018) provided a comprehensive review of the role of thyroid hormones in mammalian growth, ecology and maintenance.

Determining levels of hormones non-invasively, so that it does not interfere with the natural behaviour of an individual, is crucial to the study of wild animals (Goymann, 2012). In this regard, the collection of faecal matter is ideal, because it does not require restraint or capture and thus causes minimal disturbance. In many mammalian species, thyroid hormones have been successfully measured in faecal samples [e.g. dogs and wolves, *Canis* spp., moose, *Alces alces*, killer whales, *Orcinus orca*, stellar sea lions, *Eumetopias jubatus*, northern spotted owls, *Strix occidentalis caurina* (Wasser *et al*., 2010), primate spp. (Cristóbal-Azkarate *et al*., 2016; Schaebs *et al*., 2016; Dias *et al*., 2017; Gesquiere *et al*., 2018)]. Faecal triiodothyronine (mT3) is relatively stable for several hours or days and not affected by minor fluctuations (such as diurnal patterns; Behringer *et al*., 2018).

In order for an endocrine marker to be useful for wildlife management, we need to understand how extrinsic factors, such as vegetative productivity, season and temperature and intrinsic factors, such as age and sex, affect hormone concentrations in free-ranging populations. However, studies exploring mT3 variation in large-bodied free-ranging mammals, such as African elephants, *Loxodonta africana* (henceforth elephants unless otherwise noted), are rare (Behringer *et al*., 2018). Elephants are threatened with a drastic decline in their numbers across the African continent due to habitat loss and poaching (Chase *et al*., 2016). Furthermore, climate change has led to shifts in environmental conditions, such as changes in temperature and rainfall, resulting in more frequent and extreme droughts and large-scale warming occurring every few years, referred to as El Niño (Walther *et al*., 2002; Stenseth *et al*., 2003). In fact, recent research has suggested increases in environmental temperature and risk of drought in the near future will occur if wide-ranging and drastic climate change mitigation measures are not taken (Engelbrecht *et al*., 2015). Subsequent changes in forage and water availability can have profound effects on energy balance in free-ranging mammals. Reproduction and survival of mammals, and thus population growth, is dependent on sufficient energy being available (Dierenfeld, 1994). Therefore, understanding the energetic demand and condition of a long-lived and slow reproducing animal, such as the African elephant, is important for managers and conservation biologists tasked with ensuring the species survives in the future.

Only three published studies have investigated factors affecting thyroid hormones, but in small numbers of captive African and Asian elephants, *Elephas maximus* (Brown *et al*., 2004, 2007; Chave *et al*., 2019). In a fourth study, thyroid hormone concentrations of African elephants that had been culled were assessed (Brown *et al*., 1978). A fifth study reported that T3 can be successfully measured in the faeces of African elephants but did not investigate factors affecting mT3 concentrations (Wasser *et al*., 2010).

As previously stated, thyroid hormones correlate with caloric intake (Eales 1988; Cristóbal-Azkarate *et al*., 2016; Behringer *et al*., 2018) and are higher when nutrient quality and quantity is high (Behringer *et al*., 2018). Protein turnover is increased with increasing thyroid hormone concentrations, which serves as an adaptive function in long-term caloric restriction; when calories are in short supply, reduction of protein turnover may ameliorate the effect of the shortage (Roth *et al*., 2002; Fontana *et al*., 2006). Thus, there should be a link between fluctuations in thyroid hormone concentrations and measures of vegetative productivity.

The NDVI reflects the density and availability of green biomass and is a good proxy for forage quality (Goward and Prince, 1995; Pettorelli *et al*., 2011; Pokharel *et al*., 2018). NDVI has been used previously to assess ecological variation in both Asian and African elephant habitats (Wittemyer *et al*., 2007; Pokharel *et al*., 2018). African elephants spatially track peak NDVI across the landscape when foraging (Branco *et al*., 2019) and diet shifts in elephants, as measured by stable isotopes in elephant hair, are related to variation in NDVI (Cerling *et al*., 2006; Wittemyer *et al*., 2008). In Asian elephants, high NDVI and concurrent higher faecal nitrogen and protein content (as a proxy for dietary protein) were related to declining faecal glucocorticoid metabolite concentrations, a measure of stress (Pokharel *et al*., 2018). Further, elephant movement patterns, home range size and fine-scale selection of habitats within their home range have all been linked to variation in NDVI (Wittemyer *et al*., 2008; Cerling *et al*., 2009; Young *et al*., 2009; Wall *et al*., 2013). Additionally, high NDVI values are related to increased conception rates in elephants, as well as increased survival of young, especially in dry savannah habitats (Rasmussen *et al*., 2006; Trimble *et al*., 2009). Therefore, high NDVI may reflect increased thyroid hormone concentrations in elephants.

A further factor that interacts with metabolic activity, and therefore thyroid hormone concentrations, is environmental temperature. The thermoneutral zone (Porter and Kearney, 2009) of African elephants has, to our knowledge, not been defined. Weissenböck *et al*. (2010) have suggested that temperatures between 9 C–20.3 C lie within African elephants’ thermoneutral zone. African elephants start using evaporative cooling mechanisms at 12 C (Dunkin *et al*., 2013), much earlier than the suggested upper limit of 20.3 C (Weisseböck *et al*., 2010). Further, free-living animals experience a shift towards lower temperatures of the thermoneutral zone overall (Mitchell *et al*., 2018). Therefore, environmental temperatures are not necessarily representative of animals’ actual surface temperatures (Mitchell *et al*., 2018). In fact, elephants hardly experience thermoneutrality, but adjust physiological and behavioural mechanisms in response to unfavourable temperatures, allowing them to thrive in environments which are substantially hotter than elephants’ core body temperature (Weissenböck *et al*., 2010; Mole *et al*., 2016; Mitchell *et al*., 2018).

Elephants in South Africa are exposed to a wide range of temperatures, from sub-zero degrees during winter nights to above 40 C during summer days. High environmental temperatures are linked to reduced concentrations of thyroid hormones (Behringer *et al*., 2018) as the need for thermogenesis is reduced. Research on captive African and Asian elephants has shown a relationship between thyroid hormones and temperature, with decreased thyroid hormone concentrations during warmer summer months (Brown *et al*., 2007). In wild populations, as forage quality and dietary protein are likely to be highest when NDVI is high, we would expect a positive effect of NDVI on T3 and a negative effect of temperature on T3. Where high NDVI and high temperature occur simultaneously, an interaction between the two factors could affect T3 concentrations. In many areas, high average monthly temperatures, as well as higher average NDVI, are associated with the summer season. Season has not been found to affect T3 concentrations of captive female African or Asian elephants (Brown *et al*., 2004). However, in 12 captive male African elephants, but not Asian elephants, there was an effect of season, with lower T3 concentrations occurring during the warmer summer months (Brown *et al*., 2007). Additionally, lower T3 concentrations were reported in 51 culled African elephants in Uganda during the dry season, which authors suggested to be due to a decline in food quality, as this region did not exhibit large temperature changes between seasons (Brown *et al*., 1978).

Growth and sex can also affect T3 concentrations. Growth is related to an increased demand for catabolism of proteins and carbohydrates and is energetically demanding (Behringer *et al*., 2018). In accordance with growth being energetically demanding, thyroxine (T4) and T3 concentrations in African and Asian male elephants decrease with increasing age, as growth slows (Brown *et al*., 2007; Chave *et al*., 2019). There may be an effect of sex on thyroid hormones, as elephant bulls in musth (a state of heightened aggressive and sexual behaviour in bulls) had decreased concentrations of thyroid hormones (Brown *et al*., 2007; Chave *et al*., 2019). However, there were no age or sex-related differences in thyroid hormone concentrations of 14 male and 37 female wild African elephants, aged between 1–60 years (Brown *et al*., 1978). Generally, variation in T3 concentrations are not well understood within and across species, but possibly depend on sex-related requirements such as fecundity, lactation and pregnancy (Behringer *et al*., 2018).

Here we assessed factors affecting mT3 concentrations in a large population of elephants in Madikwe Game Reserve, South Africa (henceforth Madikwe) to better understand the use of mT3 as a potential endocrine marker for wildlife management. We examined the effect of availability and density of green biomass using NDVI, temperature, sex and age on elephant mT3 concentrations. We predicted a negative interaction between NDVI and environmental temperature on concentrations of mT3, because high environmental temperatures coincide with high values of NDVI at our study site. Further, we predicted that mT3 concentrations would be higher in young- compared to adult elephants, due to increased energetic demands of growth. Due to previously mixed findings on the effect of sex on T3 concentrations, we had no prediction for sex. Although season has previously been indicated to affect T3 concentrations (Brown *et al*., 1978; Brown *et al*., 2007), we did not investigate this in our study as dry and wet season closely related to values of NDVI and temperature, and because water was provided year-round at our study site.

## Materials and methods

### Study site

Madikwe is a fenced reserve, ~680 km^2^ large, and managed by a state/private/communal partnership. Between 1992 and 1999, a founding population of 228 elephants was reintroduced to Madikwe (Hofmeyr *et al*., 2003). At the time of the study, the reserve contained an estimated 1348 ± 128 elephants (July 2017, North West Parks Board, P. Nel, pers. comm.) or 1.9 elephants per km^2^. The reserve contains 33 lodges, each with a waterhole, as well as several large artificial dams, and the Marico River on the eastern side, all providing water year-round.

### Data and sample collection

All data were collected using non-invasive methodologies and received permission from the South African North West Parks Board as well as ethical clearance from Liverpool John Moores University (NK_IS/2016-6). This research adhered to the Association for the Study of Animal Behaviour (ASAB) guidelines for the ethical treatment of animals (ASAB, 2018).

Faecal samples were collected between April 2016 and June 2017 throughout the reserve from unidentified individuals, as well as elephants with individual identification (ID), upon watching an elephant defaecate. Individually identified elephants were recognized based on morphological features such as holes and notches in their ears, wrinkles across the face, scars and other features such as lack of tusk/s. Samples were collected from the road, once all elephants moved away and average time lag (± SD) from observing an elephant defaecate to sample collection was 16 min (± 12 min). We followed previously published protocols (Ganswindt *et al*., 2010a, b) collecting ~50 g of faecal matter in sterile vials in a cooler box on ice and transferred these to a freezer at −18 C within 4 h of collection. We recorded age (adult, juvenile or calf; Elephantvoices.org; Lee and Moss, 1986), sex, time and date, as well as ID, if known, of the defaecating individual on a Lenovo TAB 2 A8-50F tablet. Further, we extracted hourly temperature (www.meteoblue.com) during each hour a sample was collected in order to test for an association between ambient temperature and mT3 concentrations. Female elephants were identified by their genitalia, mammary glands, as well as an angled forehead, while males were identified by their genitalia, rounded forehead, wider skull and absence of mammary glands (elephantvoices.org). We did not include any samples of elephant bulls exhibiting signs of musth (Ganswindt *et al*., 2010a).

To calculate monthly mean NDVI, we used Moderate Resolution Imaging Spectrometer (MODIS) daily NDVI (Lunetta *et al*., 2006) images and Landsat 8 (Roy *et al*., 2014) eight-day composites. Images were downloaded using Google Earth Engine on the 8th of January 2018 with a 250 m and 30 m resolution for MODIS and Landsat 8 images, respectively.

We calculated monthly mean temperatures (°C) by extracting daily mean temperatures from historical records online. Daily means had been calculated from temperatures recorded at three-hour intervals (starting at 12 am) throughout each day (https://worldweatheronline.com/madikwe-weather-history/north-west/za.aspx). Mean monthly temperature ranged from 16 C to 30 C.

### Faecal triiodothyronine (mT3) extraction and analysis

T3 extraction and analysis was carried out at the Endocrine Research Laboratory, University of Pretoria, South Africa. Faecal matter was lyophilized, sieved through a mesh to remove undigested faecal matter, and between 0.050–0.055 g of the remaining faecal powder was extracted with 3 ml 80% ethanol in water. The suspension was vortexed (15 min) and centrifuged (10 min at 1500 g) and the supernatant transferred to a microcentrifuge tube. Using a T3 EIA Kit from Arbor Assays (Cat no.: K056; Ann Arbor, Michigan 48108-3284 United States), total T3 in diluted extracts (1:50 or 1:100 in aqueous buffer) was analysed. In brief, 0.1 ml aliquots of standards (range = 78–5000 pg/ml), quality controls and diluted faecal extracts were pipetted in duplicate into microtiter plate wells. A T3-peroxidase conjugate and an antiserum (raised in sheep) was added and the plates were incubated for 2 h. After this, the plate was washed four times and substrate added. After incubation for 30 min, the reaction was stopped, and absorbance was measured at 450 nm. This EIA has been biologically validated for determining mT3 concentrations of elephants by comparing individual mT3 levels of temporally injured individuals. In a study by Ganswindt *et al*. (2010b), body condition was used as a proxy for health and nutrition status, as the two monitored individuals showed a distinct decrease in body condition due to a foot injury causing temporary lameness. For the EIA validation, the temporally low body condition scores recorded should be associated with comparatively lower individual mT3 concentrations. At lowest recorded body condition, related mT3 levels were 0.18 μg/g dry weight (DW) and 0.30 μg/g DW, respectively, for the two bulls. Following recovery, the condition of both bulls improved progressively and related mT3 levels peaked at 0.84 μg/g DW and 0.63 μg/g DW, respectively. Sensitivity of the assay at 90% binding was 37.4 pg/ml, with a detection limit of the assay of 46.6 pg/ml (Arbor Assays information). Intra-assay coefficient of variation (CV), determined by repeated measurements of high- and low-value controls, is 5.5% and 6.7%, respectively. Inter-assay CV, determined by repeated measurements of high- and low-value controls, is 11.6% and 14.7%, respectively (Arbor Assays information). We measured an inter-assay CV, using repeated measurement of high value control, of 10.9%.

### Data analysis

All data were analysed in R v.3.4.1 (R Core Team, 2000). We assessed factors using variance of inflation factor (VIF) analysis (Fox and Monette, 1992) in the car package (Fox and Weisberg, 2011) to rule out collinearity, using a cut-off value of 3. All VIF values were below 2.3. Hourly temperature during sample collection was not associated with mT3 concentrations in faecal samples (Pearson’s correlation: r (186) = −1.6, *P* = 0.112); therefore, we used mean monthly temperature for the analyses.

We analysed samples of unidentified as well as identified individuals with an a priori generalized linear (mixed) model with a gamma error structure and log link because data were not normally distributed and only resembled a normal distribution with a log10 transformation. Using the ‘glm’ command in the R base code, we ran the following model for unidentified individuals:

$$\begin{align*} \textrm{glm} (\textrm{formula} =&\ \textrm{mT}3 \sim \textrm{NDVI}^{\ast} \textrm{Temperature} + \textrm{Age} + \textrm{Sex}, \textrm{data}\\ =&\ \textrm{Data}, \textrm{family} = \textrm{Gamma}(\textrm{link} = ``\textrm{log}\textrm{''}))\end{align*}$$

And using the ‘glmer’ command (lme4 package, Bates *et al*., 2007), we ran the following model for the identified individuals:

$$\begin{align*} \textrm{glmer} (\textrm{formula} =&\ \textrm{mT}3 \sim \textrm{NDVI}^{\ast} \textrm{Temperature} + \textrm{Age} + \textrm{Sex}\\ &+ (1|\textrm{ID}), \textrm{data} = \textrm{Data}, \textrm{family}\\ =&\ \textrm{Gamma} (\textrm{link} = ``\textrm{log}\textrm{''}))\end{align*}$$

As our initial models, using unstandardized data, reported convergence warnings and large eigenvalues, we scaled and centred NDVI and temperature data to rectify these issues and improve model fit. To scale data, we calculated the mean and standard deviation of the entire vector and then scaled each element by subtracting the mean, before dividing it by the vector’s standard deviation. We then centred the variable around zero. This method does not affect statistical inference in regression models and further eases interpretation of effects where parameters have different scales (e.g. a large range of temperatures compared to NDVI values ranging between −1 to 1, Schielzeth, 2010). For the unidentified individuals, we bootstrapped confidence intervals with 1000 iterations using the package boot (Canty and Ripley, 2018) because of possible non-independence in the data due to potential pseudo-replication. Significance was assigned where the 95% confidence intervals did not cross zero.

To control for the relatively small sample size of the known individuals’ data set (*n* = 43), we used a Kenward–Roger approximation, fitted with restricted maximum likelihood estimation (Kenward and Roger, 1997; Luke, 2017) with the afex package (Singmann *et al*., 2018), to obtain *P*-values for our fixed effects. Significance was assigned at *P* ≤ 0.05. We plotted all graphs with unscaled data, using the packages effects (Fox, 2003) and ggplot2 (Wickham, 2016).

## Results

### Unidentified individuals

**Figure 1:**
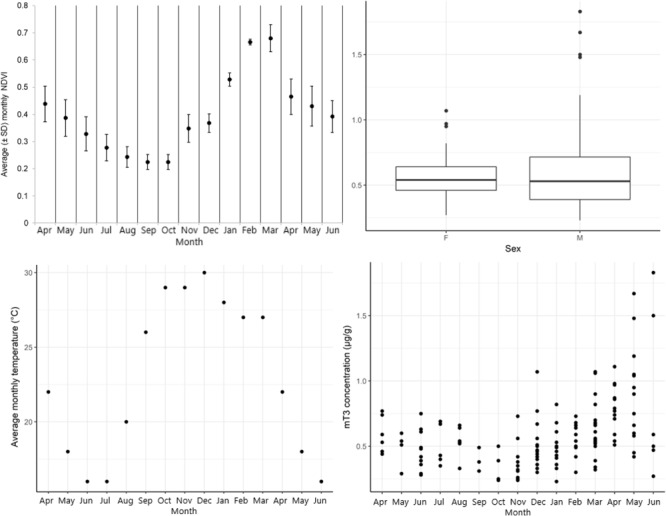
Descriptive statistics of average (± SD) monthly NDVI values each month (top left), average faecal thyroid metabolite (mT3) concentrations (μg/g DW) of unidentified female (F) and male (M) African elephants, *Loxodonta africana* (*n* = 145 samples, top right), average monthly temperature each month (bottom left) and raw values of mT3 concentrations each month (bottom right). NDVI can range from −1 to 1, where lower values indicate less green vegetation. All data from April 2016 to June 2017, collected in Madikwe Game Reserve, South Africa.

**Figure 2:**
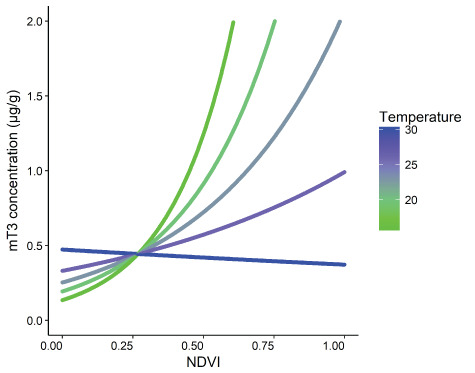
The interaction between monthly mean NDVI and monthly mean temperature on faecal thyroid metabolite (mT3) concentrations (μg/g DW) of unidentified free-ranging African elephants, *Loxodonta africana* (*n* = 145 samples), in Madikwe Game Reserve.

**Figure 3:**
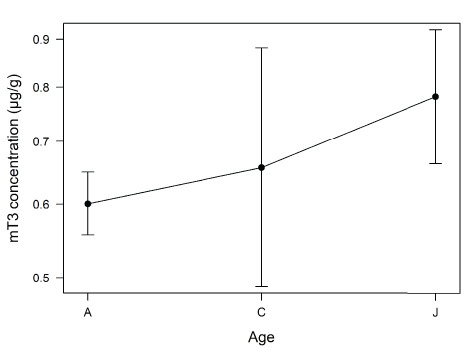
Effect of age on faecal thyroid metabolite (mT3) concentration (μg/g DW) of unidentified adult (A, *n* = 118 samples), calf (C, *n* = 6 samples) and juvenile (J, *n* = 21 samples) free-ranging African elephants, *Loxodonta africana* (*n* = 145 samples), in Madikwe Game Reserve. Error bars represent 95% confidence intervals.

We collected a total of 145 samples (mean (± SD) per month = 9 ± 5) from unidentified individuals (Fig. 1). These were 48 and 70 samples from adult females and males, respectively, 7 and 14 samples from juvenile females and males, respectively and 2 and 4 samples from female and male calves, respectively. Overall mT3 concentrations ranged from 0.23 μg/g to 1.83 μg/g DW with an overall mean (± SD) of 0.59 (± 0.27) μg/g DW [0.56 (± 0.16) for females and 0.6 (± 0.32) for males]. We found a significant negative interaction effect of NDVI and temperature on mT3 (Table 1, Fig. 2). Juvenile elephants had significantly higher mT3 concentrations compared to adult elephants (Table 1, Fig. 3). Sex did not have a significant effect on mT3 concentrations, as 95% confidence intervals crossed zero (Table 1).

**Table 1:** Bootstrapped 95% confidence intervals of a GLM of effects on faecal T3 (mT3) concentrations of unidentified African elephants, *Loxodonta africana*, in Madikwe Game Reserve. Fixed effects’ estimates and standard errors (SEs) are from the model summary and 95% confidence intervals are from a non-parametric bootstrap with 1000 iterations. Significant effects shown in bold

Fixed effect	Level	Estimate (± SE)	Level (reference level: comparison level)	95% Confidence intervals
Intercept		−3.446 ± 077		
**NDVI**		9.793 ± 2.09	NDVI	3.859 to 9.220
**Temperature**		0.090 ± 0.03		0.032 to 0.096
**Age** (adult)	Calf	0.089 ± 0.15	Adult: calf	−0.093 to 0.225
	Juvenile	0.263 ± 0.09	Adult: juvenile	0.054 to 0.317
			Juvenile: calf	−0.355 to 0.113
Sex (female)	Male	−0.053 ± 0.06	Female: male	−0.061 to 0.059
NDVI^*^ temperature		−0.334 ± 0.08	NDVI^*^ temperature	−0.330 to −0.129

### Identified individuals

**Figure 4:**
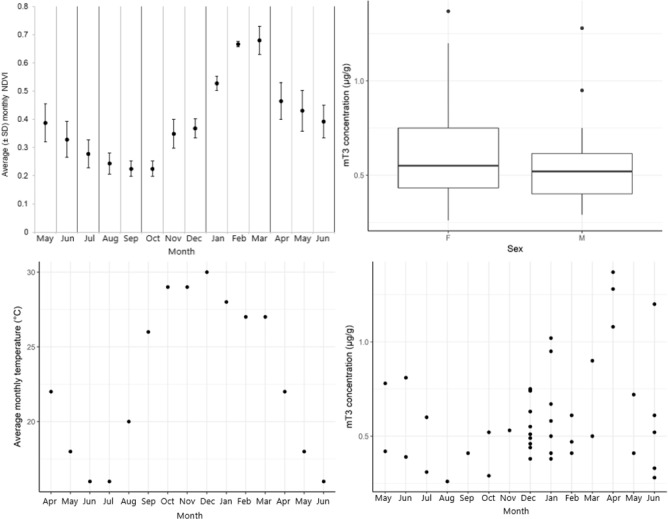
Descriptive statistics of average (± SD) monthly NDVI values each month (top left), average faecal thyroid metabolite (mT3) concentrations (μg/g DW) of individually identified female (F) and male (M) African elephants, *Loxodonta africana* (*n* = 43 samples, top right), average monthly temperature each month (bottom left) and raw values of mT3 concentrations each month (bottom right). NDVI can range from −1 to 1, where lower values indicate less green vegetation. All data from April 2016 to June 2017, collected in Madikwe Game Reserve, South Africa.

**Figure 5:**
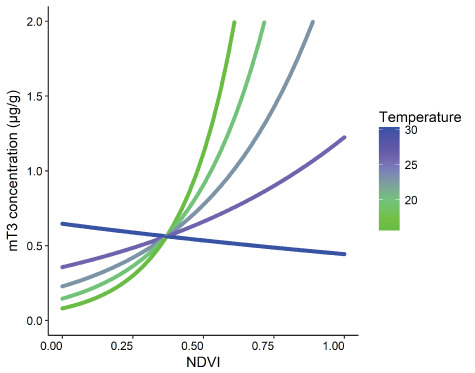
Interaction effect of monthly mean NDVI and monthly mean temperature on faecal thyroid metabolite (mT3) concentration (μg/g DW) of 13 identified free-ranging African elephants, *Loxodonta africana* (*n* = 43 samples), in Madikwe Game Reserve, South Africa

We collected 43 samples (mean ± SD per month = 3.3 ± 1.9) from 13 identified individuals (one male calf, three juvenile females, three juvenile males and five adult females, all in four different cow-calf groups and a solitary adult male, Table 2, Fig. 4). Overall mT3 concentrations ranged from 0.26 μg/g to 1.37 μg/g DW with an overall mean (± SD) of 0.6 (± 0.27) μg/g DW. The random effect of ID had a variation (± SD) of 0.027 ± 0.17. We found a significant negative interaction effect of NDVI and temperature on the concentration of mT3 metabolites (Table 3, Fig. 5). Neither sex nor age had a significant effect on mT3 concentrations of identified individuals (Table 3).

## Discussion

Conservation management decisions often require an understanding of the influence of anthropogenic pressures on wildlife health. Endocrine markers, such as thyroid hormones, have great conservation value for quantifying and monitoring the energetic state of animals. In order for such markers to be utilized effectively as a wildlife management tool, they need to be applied in wild populations, where factors influencing varying concentrations can be determined. Our study assessed the effect of ecological and intrinsic factors and found a significant negative interaction effect of NDVI and temperature, as well as an effect of age, on mT3 concentrations in unidentified elephants. In the sample of identified elephants, we found a significant negative interaction effect of NDVI and temperature. The effect of age on mT3 concentrations in unidentified elephants was in line with published literature of a range of species (Behringer *et al*., 2018; Chave *et al*., 2019). As predicted, increasing NDVI was positively related to mT3 concentrations at low temperatures, while increasing temperature weakened the effect of NDVI. Sex did not affect mT3 concentrations in our study in either data set investigated. To our knowledge, this presents the first findings of factors affecting mT3 concentrations in free-ranging elephants.

**Table 2:** Descriptive statistics of faecal thyroid hormone (mT3) concentrations of 13 individually identified African elephants, *Loxodonta africana*, in Madikwe Game Reserve, South Africa. Concentrations are in μg/g DW. ID number of individuals, their age and sex are presented [with overall mean (± SD) mT3 concentrations] and a breakdown of number (*n*) of samples collected for each individual

ID	Sex	Age	Number of samples per individual
1: 0.71 (± 0.14)	Female	Adult 0.65 (± 0.3)	2
2: 0.51 (± 0.22)	0.63 (± 0.28)		4
3: 0.58 (± 0.25)			8
4: 0.45 (± 0.10)			2
5: 1.12 (± 0.30)			3
6: 0.70 (± 0.25)		Juvenile 0.59 (± 0.22)	5
7: 0.50 (± 0.01)			2
8: 0.41 (± 0.04)			2
9: 0.48 (± 0.08)	Male 0.56 (± 0.26)	Adult 0.48 (± 0.08)	2
10: 0.58 (± 0.36)		Juvenile 0.48 (± 0.26)	6
11: 0.78 (± 0.25)			2
12: 0.40 (± 0.12)			3
13: 0.65 (± 0.14)		Calf 0.65 (± 0.14)	2

We found that NDVI and temperature had a negative interaction effect on mT3 concentrations (Figs 2 and 5). As temperature increased, the positive effect of NDVI on mT3 concentrations weakened, with temperatures above 25°C reducing the effect of NDVI by ~50% (Figs 2 and 5). In this study, NDVI was used as a proxy for dietary protein and forage quality (Goward and Prince, 1995; Pettorelli *et al*., 2011; Pokharel *et al*., 2018) and was positively related to concentrations of mT3, aligning with previous literature on a range of mammals (Behringer *et al*., 2018). It is possible that we found such a strong positive relationship between NDVI and mT3 concentrations at Madikwe because the energy gain from the increase in forage quantity was not offset by energy expended in search of scarce water sources. In Madikwe, pumped water supplies were provided across the reserve throughout the year and thus, the location of water was unlikely to constrain elephant foraging patterns. In other areas, where no artificial water is provided or which are unfenced, the location of water sources influences elephant foraging and ranging patterns (Dunkin *et al*., 2013; Bastille-Rousseau and Wittemyer, 2019) and mT3 concentrations may be even lower during times of low NDVI and water availability. The effect of temperature on metabolic rate is well known. Endotherms reduce their metabolic rate at high environmental temperatures (Speakman and Król, 2010) and previous research has suggested that temperature is related to mT3 alterations in elephants (Brown *et al*., 2007). The negative effect of temperature on mT3 concentrations will become increasingly important considering the changing global climate. Higher environmental temperatures overall, especially during times of reduced nutrient availability, could negatively affect mT3 concentrations directly through temperature-related effects, as well as indirectly, through effects on water and nutrient availability caused by climate events such as El Niño (Stenseth *et al*., 2003) and climate change (Engelbrecht *et al*., 2015; Mitchell *et al*., 2018). For example, under low climate change mitigation scenarios, southern Africa has recently been suggested to suffer from 4–6°C increases in annual maximum temperatures, increased risk of droughts and wildfires and decreased rainfall in the near future (Engelbrecht *et al*., 2015). Negative effects on mT3 concentrations related to such future changes could, in turn, affect growth and reproduction of elephant populations (Milewski, 2000; Shannon *et al*., 2008). Our findings emphasize the importance of considering environmental temperature when assessing energetic condition of free-ranging and captive elephants and, given the similarities with a range of published studies, other species of mammals (Brown *et al*., 2007; Behringer *et al*., 2018).

It is widely accepted that BMR, which closely correlates with thyroid hormones across species (Bianco *et al*., 2005; Mullur *et al*., 2014), declines with age (Keys *et al*., 1973). For example, previous studies have shown that mT3 concentrations are elevated in juvenile mammals and this is thought to be due to energetic demands of growth (Behringer *et al*., 2018). Our results from unidentified elephants support those findings and from studies on captive elephants (Brown *et al*., 2007; Chave *et al*., 2019), as we found that juveniles had significantly elevated mT3 concentrations compared to adults. However, we found similar mT3 concentrations in calves and adults. In cattle, high ambient temperatures slow calf growth (e.g. Kadzere *et al*., 2002) and as samples from calves were collected between October 2016 and April 2017, months during which environmental temperatures were high (Fig. 1), it is possible that this affected mT3 concentrations. Further research is needed to fully understand the effect of high temperatures on calves and, given our small sample size, we cannot draw a robust conclusion. The lack of an effect of age in our identified elephants, on the other hand, may be due to the small number of samples of adult and juvenile elephants (*n* = 6 each). Overall, the mean (± SD) concentration of mT3 in juveniles was 0.58 ± 0.26 μg/g DW and in adults was 0.63 ± 0.29 μg/g DW. The exact causes of the decline in thyroid hormones with increasing age are not well understood and further research is required to understand the flexibility of metabolic strategies in wild animals (Behringer *et al*., 2018).

Our study did not find an effect of sex on elephant mT3 concentrations and future studies should assess this across reproductive states, for example by comparing mT3 concentrations of known individuals during pregnancy, lactation or musth. For example, female baboons had lower mT3 concentrations during pregnancy or lactation (Gesquiere *et al*., 2018), while wild howler monkeys had increased concentrations of mT3 during pregnancy and lactation (Dias *et al*., 2017). Unfortunately, we did not collect data on reproductive state and were therefore unable to include these factors in our analyses. However, three of the identified females were nursing a calf throughout the study period and therefore fluctuations in their mT3 concentrations could not be related to a change in reproductive state, for example from pregnant to lactating. Repeated sampling of known individuals may reveal fine-scale fluctuations of mT3 concentrations related to such stages.

During fasting and low food availability, protein turnover is reduced and concentrations of T3 are low (St Aubin *et al*., 1996; Oritz *et al*., 2010). Elephant bulls in musth reduce their food intake and lose body condition (Poole 1987, 1989), and musth has been linked to reduced thyroid hormone concentrations (Chave *et al*., 2019). As shown for the EIA validation for the research presented here, low body condition scores were associated with low concentrations of mT3 in elephant bulls. In those bulls, there was a decrease in mT3 concentrations of 366% and 110% from individual baseline values, respectively. Interestingly, the animal that showed the more prolonged and severe injury not only showed a higher impact on body condition (down to 2 out of 4) but also the lowest mT3 levels (0.18 μg/g DW) during this time, compared to the other animal monitored (body condition down to 3 out of 4, mT3 levels: 0.3 μg/g DW). So, it could be speculated that in these cases severity of injury impacted on body condition, which might be reflected in the different quality of response with regards to mT3 levels. However, a low body condition score is the result of a lack of sufficient nutrition after a longer period of time and thus may not provide an early warning to facilitate a timely management response. Changes in hormone concentrations can be detected at an earlier stage than changes in body condition and can, therefore, be considered as more proactive measures of individuals’ energetic condition.

**Table 3:** Estimate and SE of a GLMM on faecal T3 (mT3) metabolite concentrations of individually identified African elephants, *Loxodonta africana*, in Madikwe Game Reserve and degrees of freedom (df), F- and *P*-value of the GLMM as assessed with a Kenward–Roger approximation. Significant effects are shown in bold

Fixed effect	Level	Estimate (± SE)	df	F	*P*-value
Intercept		−0.443 ± 0.16			
**NDVI**		0.234 ± 0.08	35.29	5.10	0.03
Temperature		−0.123 ± 0.09	35.73	2.01	0.17
Age (adult)	Calf	0.087 ± 0.47	10.45	0.07	0.93
	Juvenile	−0.069 ± 0.24			
Sex (female)	Male	0.013 ± 0.25	9.24	0.00	0.95
NDVI^*^ temperature		−0.315 ± 0.11	33.60	4.61	0.04

The use of endocrine markers by conservation biologists is only possible given an understanding of how those hormone concentrations are affected by various environmental and intrinsic factors. As such, this study, to our knowledge, is the first to provide information on the effects of NDVI and temperature on mT3 concentrations. We found that elephants may be most energetically constrained, and therefore more vulnerable to external pressures, during periods of low NDVI and high environmental temperature. In Madikwe, this was the case during the months of September and October. Managers trying to conserve populations should be aware of the interactive effects of environmental stressors. Recent research has shown that elephants most strongly selected habitat close to permanent water sources (Bastille-Rousseau and Wittemyer, 2019). However, where human activity increased, elephants showed stronger selection for woodland savannah and weaker selection for habitat productivity and permanent water sources (Bastille-Rousseau and Wittemyer, 2019). This suggests that anthropogenic pressures may become increasingly important in shaping elephant habitat use and although elephants are flexible in their selection strategies, this may add to the energetic constraints of elephants. Further, given the more extreme temperatures in recent years related to events such as El Niño (Stenseth *et al*., 2003), predicted increases of average temperatures and more frequent droughts related to climate change (Engelbrecht *et al*., 2015), extreme environmental conditions may require managers to consider more targeted management strategies, such as provision of water sources and additional forage.

## Conclusion

We provided first insights into mT3 concentrations of free-ranging African elephants, finding that NDVI in interaction with temperature and age were related to mT3. Understanding mT3 concentrations in elephants and how they are affected by environmental conditions as well as by different life-history stages can help wildlife managers in using this endocrine marker more effectively. Such studies will enable managers to predict nutritional needs of elephants more accurately and identify times when elephants are more vulnerable to additional external stressors.
